# Evaluating intrinsic and non-intrinsic cancer risk factors

**DOI:** 10.1038/s41467-018-05467-z

**Published:** 2018-08-28

**Authors:** Song Wu, Wei Zhu, Patricia Thompson, Yusuf A Hannun

**Affiliations:** 1 Department of Applied Mathematics and Statistics, Stony Brook University, Stony Brook, NY 11794 USA; 2 Stony Brook Cancer Centre, Stony Brook University, Health Sciences Centre, Stony Brook, NY 11794 USA; 3 Department of Pathology, Stony Brook University, Health Sciences Centre, Stony Brook, NY 11794 USA; 4 Department of Medicine, Stony Brook University, Health Sciences Centre, Stony Brook, NY 11794 USA

## Abstract

Discriminating the contribution of unmodifiable random intrinsic DNA replication errors (‘bad luck’) to cancer development from those of other factors is critical for understanding cancer in humans and for directing public resources aimed at reducing the burden of cancer. Here, we review and highlight the evidence that demonstrates cancer causation is multifactorial, and provide several important examples where modification of risk factors has achieved cancer prevention. Furthermore, we stress the need and opportunities to advance understanding of cancer aetiology through integration of interaction effects between risk factors when estimating the contribution of individual and joint factors to cancer burden in a population. We posit that non-intrinsic factors drive most cancer risk, and stress the need for cancer prevention.

## Abstract (web summary)

Understanding the contributions of extrinsic and intrinsic factors on cancer risk is fundamental in determining the intervention and prevention strategies to tackle cancer. Here the authors provide a review of the different factors impacting cancer risk and discuss the limitations of different approaches in evaluating the relative contributions of these factors.

## Introduction

The past few decades have seen significant progress in our understanding of cancer aetiology as well as advances in early detection, treatment, and prevention^1–3^, which have led to declining cancer mortality in the industrialized world. Despite this progress, certain cancers continue to increase in different parts of the world due, in part, to longer lifespans and changing patterns of cancer risk factors^4^. This includes the first evidence of impacts of the obesity epidemic on cancers^5^. Furthermore, significant gaps in age-adjusted cancer incidence rates for nearly all cancers across different regions of the world suggest that much of cancer risk is due to causes other than unmodifiable intrinsic DNA replication errors common to all humans which we define as the ‘intrinsic risk’^6^.

Extensive efforts over several decades have been directed at and continue to be expended on identifying risk factors for cancer. For several cancers, aetiology has been convincingly linked to specific environmental factors resulting in effective cancer prevention (https://www.cancer.gov/about-cancer/causes-prevention/risk), e.g., smoking and lung cancer, sun exposure and skin cancer, human papillomavirus (HPV) and cervical cancer, *Helicobacter pylori (H. pylori)* and gastric cancer, and viral hepatitis and hepatocellular cancer (HCC).

While certain external exposures have been established in cancer causation, the contribution of random errors in DNA replication has been more difficult to estimate. Two recent modelling studies suggested that over 60% of tissue cancer burden may be due to factors that are intrinsic to human cell biology and thus, not modifiable^7,8^. This conclusion has been highly contested^9–15^. Nevertheless, these provocative findings gained media attention as evidence dampening healthy behaviours for cancer risk reduction and renewed old debates on the role of modifiable factors in cancer causation among scientists. They also raised questions about the evidence that scientists use and assumptions that they make to mathematically estimate the contribution of different factors to the burden of cancer in the population setting.

Here, we briefly introduce and refine the definitions of intrinsic and non-intrinsic risk factors that have been employed in these recent works and how evidence for their effects on cancer burden in human has been obtained considering the type of study (observational or experimental). This includes a discussion of the assumptions about cancer aetiology that have been used to estimate the contribution of various factors to the burden of cancer in the human population. Through clarifying the definitions, and analyzing the cumulated models, data, and findings from historical and modern literature, we develop our position that non-intrinsic factors are the major contributors to cancer risk and thus open the door for significant prevention.

## Cancer risk factors and cancer risks

The pursuit of cancer risk factors has been instrumental in the development of both data-driven analytical approaches and theory-driven models for carcinogenesis. The former was initiated by landmark epidemiological studies of lung cancer and tobacco smoking in the 1950’s. The latter began with the modelling of carcinogenicity in animals early in the 20th century^16–18^ and subsequently in humans^19–26^, culminating in two recent contrasting models that we highlight below^8,15^.

**Fig. 1:**
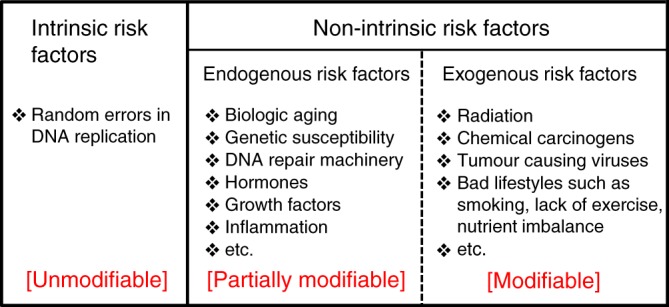
Three types of cancer risk factors. The overall cancer risk factors are divided into two mutually exclusive components: the unmodifiable intrinsic and the modifiable, at least partially, non-intrinsic risk factors. The intrinsic risk factors refer to random errors resulting from DNA replication. The non-intrinsic risk factors further consist of endogenous and exogenous risk factors depending on whether such factors are more internal or external to an individual

To facilitate the discussion and relate to recent published model-based estimates, separate categories for cancer risk factors are defined below based on their biologic nature, modifiability and use in the literature (Fig. 1):

1. Unmodifiable intrinsic risk refers to unavoidable spontaneous mutations that arise as a result of random errors in DNA replication related as a characteristic of being human. These unavoidable DNA replication processing errors occur in different organisms at different rates as a species specific, random replication error rate.
2. Non-intrinsic risk refers to factors that include: (2a) Modifiable exogenous/external factors (e.g., carcinogens, viruses, xenobiotic) and lifestyle factors (e.g., smoking, hormone therapy, nutrient intake, physical activity) that are exogenous to the host; and (2b) Endogenous factors that are partially modifiable and related to the characteristics of an individual (e.g., immune, metabolism, DNA damage response, hormone levels) and influence key aspects of cell growth control and genome integrity.

We have selected these definitions to assure better dissection of the contribution of ‘intrinsic errors’ as an unmodifiable risk factor for cancer modelled in recent studies^27,28^. Particularly, this definition of intrinsic risk implies three corollaries of high relevance to additional analyses: (1) The contribution of this unmodifiable risk to cancer incidence should be constant across populations since all humans have the same intrinsic mutation rates; (2) This contribution should also be consistent over time since the underlying mechanism is a property of the human species; and (3) the contribution of intrinsic DNA replication errors to mutational signatures should be constant across tissues and organs.

In addition, it should be noted that cancer displays a complex etiopathogenesis and that these various factors interact in tumour evolution (e.g., gene–gene or gene–environment interactions). For modelling and discussion purposes, cancer risk types have been discretized as the intrinsic risk and the non-intrinsic risk, which refers to all risk minus the intrinsic risk, or likewise, the sum of risks due to non-intrinsic factors, plus their interactions, plus the interactions between intrinsic and non-intrinsic factors. Accordingly, this definition of intrinsic risk accounts for those cancers where intrinsic error is sufficient for tumorigenesis. Thus, in deriving the intrinsic risk (so-called ‘bad luck’), one must subtract risk not only due to individual non-intrinsic factors, but also to their interactions with intrinsic factors. This group would include cases where the intrinsic factor (i.e., basal mutation rate) contributes and may be necessary but not sufficient. As an example, a specific lung cancer may arise from three driver mutations, one of which arises from an intrinsic error and two from mutagens in tobacco smoke. In this case, the intrinsic error is necessary but not sufficient for detectable invasive carcinoma to develop. From an intervention point of view, this is critical as preventing the modifiable component (i.e., smoking exposure) would still be effective in preventing cancer in these settings.

## Intrinsic risk factors

As defined above, intrinsic risk arises from the basal mutation rate operating in all dividing cells, in the absence of any non-intrinsic factors.

We have chosen to define unmodifiable intrinsic risk in this narrow way as it corresponds to a biologically intrinsic factor that causes DNA mutations in humans that is not modifiable. Thus, all humans are ‘stuck’ with this risk, unlike other sources of non-intrinsic factors that may vary between individuals.

Passage or fixation of randomly acquired mutations (e.g., single nucleotide errors, deletions and insertions) in a tissue is dependent on the survival and division of the mutated cell and its progeny. These mutations may yield “driver mutations” required for cancer development, in distinction from “passenger mutations” that do not impact cancer formation but are found commonly in cancers. A requirement for more than one driver mutation to initiate cancer increases the barrier to develop cancer with intrinsic mechanisms alone.

In 2015, Tomasetti and Vogelstein asked why different tissues exhibit dramatically disparate cancer rates. Using estimates of the number and dynamics of tissue-specific stem cells for 31 tissue types, they observed a strong correlation between estimated stem cell divisions and lifetime cancer risk at log10 scale. From their modelling work, they suggested that a significant and underappreciated component of cancer risk, as much as 64%, may be due to unmodifiable random errors in DNA synthesis or bad luck^7^. This hypothesis sparked debates^9,13^ on the nature of this correlation and its implications for causality of stem cells in cancer pathogenesis. In our work, we found that the correlation between stem cells and cancer risk does not distinguish the operation of intrinsic from non-intrinsic factors and vice versa, since many non-intrinsic factors (e.g., smoking) induce their own mutations, and the likelihood of induction and propagation of these mutations also correlates with tissue cell divisions^15^. Thus, tissues with much larger cell divisions are susceptible to higher intrinsic mutations as well as to higher mutations induced by external factors. This conclusion was supported in recent analysis by Nowak et al.^29^. Furthering the complexity of cancer risk factors, in one study, Klutstein et al.^30^ found a stronger correlation between tissue levels of DNA methylation and cancer burden. This correlation persisted even after correcting for the contribution of stem cells whereas the reverse did not hold. These authors concluded epigenetic changes, which can be influenced by exogenous and endogenous factors, and not only mutations contribute to cancer risk with a similar dependence on the number of cell divisions in a tissue. Thus, while these *correlative* studies support total tissue cell division in the observed variation between tissue-specific cancer risks, this association is correlative and only explains a part of that risk.

### Mutational signatures in human cancer reveal past events

The direct estimation of intrinsic error to cancer risk is challenged by the technical inability to truly separate intrinsic errors from the effects of non-intrinsic factors in humans. Evidence for intrinsic risk in cancer has historically relied on modelling studies of cancer development based on experimental/clinical data. The recent advent and rapid development of next-generation DNA sequencing technology has revolutionized the ability to survey genome-wide somatic mutations in cancer. Analyses of these data are providing new insight into the role of intrinsic versus non-intrinsic cancer risk factors, and in some cases, linking specific signatures to specific risk factors. Here we discuss recent work from large-scale tumour sequencing studies applied to estimating the magnitude of intrinsic risk and its contribution to human cancer.

Using genome sequence data, more than 30 distinct mutational signatures were recently uncovered in different cancers^31^. Of these, 10 can be associated, at least partially, to known mutagens. Interestingly, two signature mutations demonstrated strong positive correlations with age in most cancer types, indicating that they are acquired at a relative constant rate over the lifetime of cancer patients, regardless of tissue of origin. This pattern is most consistent with the action of an intrinsic error process, since errors arising with DNA replication during cell division would accumulate in a monotonic fashion over time. In contrast, the other signature mutations lack a consistent correlation with age, suggesting they may be acquired at different rates in life due to different influences^31^. Since all known carcinogen-specific signatures demonstrate an age-uncorrelated and tumour-specific pattern, it is reasonable to assume those with unknown causes are also a consequence of external exposures to DNA damaging agents.

Based on this segregation of signatures, the proportion of cancers driven by intrinsic risk can be calculated, as shown in Box 1, to account for no more than 10–30% of all cancer incidence^15^. Notably, a number of cancers, such as lung and skin cancer, with substantial environmental risk as determined from epidemiologic studies, also contain large percentages of non-intrinsic risk estimated from the mutational signature data (Extended Data Table 3 in ref. ^15^), supporting the validity of this approach.

### Box 1 Mutational signatures and cancer risks

Sequence analyses of large cancer genomic data suggests that for some cancers, mutations are not random and dependent on the nucleotide context around mutation sites. Such mutational signatures are sequence patterns preferably associated with specific mutagens and are regarded as ‘fingerprints’ left on cancer genomes by different mutagenic processes. For example, because UV radiation usually induces formation of covalent links between two adjacent pyrimidines, C>T mutations due to UV occur mainly at dipyrimidine sequences^81^. More than 30 distinct signatures have been identified so far, and several of them have been mechanistically associated with known risk factors such as UV radiation and smoking. A few signatures demonstrate strong positive correlations with age in the majority of cancers, suggesting they likely arise from some fundamental tissue-independent and constant intrinsic biological processes, such as replicative errors in cell divisions.

These data can be used to estimate (1) the percentage of mutations due to intrinsic factors, and (2) the intrinsic risk. Suppose the percentage of intrinsic mutations in a specific cancer is *p*, and *n* driver mutations are needed for cancer onset. Intrinsic risk is then defined as the probability of incurring the *n* driver mutations with intrinsic mechanism only and can be calculated as

$${\mathrm{Intrinsic}}\,{\mathrm{risk}} = \left( {\mathrm{cancer}\,{\mathrm{incidence}}} \right) \ast p^n.$$

For example, when *p* = 0.5, *n*=3, and cancer incidence = 1%, the intrinsic risk is then 1% * 0.125 = 0.00125. Similarly, using the binomial distribution, one can compute risk due to extrinsic factors alone, and risk due to the interactions between intrinsic and extrinsic factors. It should be noted that not all carcinogens are mutagens, and therefore would not leave signatures on genomes. However, it has been observed that many cancers known to have substantial environmental risk proportions, such as breast cancer, colorectal cancer and melanoma, all harbour large percentages of total extrinsic mutational signatures. More interestingly, for cancers such as acute myeloid leukemia (AML) and acute lymphocytic leukemia (ALL), that do not have strong epidemiology support for their environmental causes, their intrinsic risks calculated from mutational signatures are relatively high.

The current non-intrinsic cancer risk estimates from the mutational signature data assumes that the intrinsic and extrinsic mutagenic mechanisms have the same probability of inducing mutations in cancer driver genes. Biased estimations may arise if such an assumption is unattainable. In addition, more than 900 chemical agents have been evaluated by the International Agency for Research on Cancer (IARC), of which more than 400 have been identified as carcinogenic, probably carcinogenic, or possibly carcinogenic to humans^124^; however, mutational signatures for these mutagens remain largely unidentified. Uncovering these would further improve the accuracy of the estimated cancer risk distributions.

### Modelling of contribution of intrinsic mutations

Several studies have attempted to estimate the number of driver mutations required for the development of an invasive carcinoma. The emerging consensus is that at least three hits are necessary for solid tumours and fewer for haematologic malignancies. The historical development of this work is shown in Box 2.

Replication error rate is a critical parameter in modelling intrinsic cancer risk in human cells, and the unrepaired error rate has been estimated at ~5 × 10^−10^ per nucleotide site per cell division^32^. This corresponds approximately to three new mutations per genome per cell division. Replication error rates between different cell types in an organism are roughly constant given the fundamental nature of the replication process. For proto-oncogenes, gain-of-function mutations typically occur at specific sites that increase action of the target protein (e.g., JAK2^V617F^ or KRAS^G12V^). In contrast, loss-of-function mutations can occur at multiple sites whereby numerous mutational events promote gene loss or dysfunction (e.g., P53 mutations). Thus, the probability of mutating at least one cancer relevant gene is larger than the somatic mutation rate of one nucleotide. For example, if 20 mutable sites correspond to one cancer relevant hit, the probability of that hit would be 1 × 10^−8^ per cell division.

Based on these and related data, we developed a discrete stochastic multistage cancer stem cell model, with the model parameters (number of stem cells, intrinsic mutation rate, and the generations of symmetric versus asymmetric divisions) estimated from the most recent literature^15^. Once the intrinsic risk due to replication errors was computed, the difference between the model estimation and the observed epidemiological cancer incidence provided an estimate of the non-intrinsic risk (residual risk). These results suggested that cancer risk due to intrinsic factors alone is very low for cancers requiring more than two hits, consistent with other independent analyses including observational studies and a mutational signature study. Based on these data, we concluded that intrinsic risk explains at most 10–30% of all cancers^15^.

More recently, Tomasetti et al.^8^ published a new estimate of the proportion of cancer driver gene mutations due to intrinsic factors. For 32 cancers examined, they concluded that 66% of mutations were attributable to intrinsic causes. A major cornerstone of this recent work was the calculation of the intrinsic risk as the amount of risk that remains after subtracting effects of known environmental and hereditary factors. That is, the percentage of mutations due to intrinsic factors was computed as:

$$({\mathrm{Percent}}\,{\mathrm{due}}\,{\mathrm{to}})\,{\mathrm{Intrinsic}} = 1 - {\mathrm{known}}\,{\mathrm{environmental}} - {\mathrm{known}}\,{\mathrm{hereditary}}$$

**Fig. 2:**
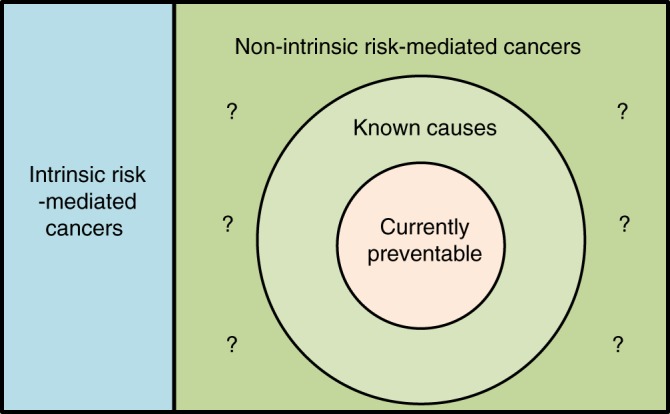
This diagram illustrates the relationship between intrinsic and non-intrinsic risks, as well as preventable cancer and overall cancer burden. One can see that by ignoring the unknown non-intrinsic risk (area marked with?), the estimated intrinsic risk in ref. ^8^ is inflated as the true intrinsic risk (blue region) plus the unknown non-intrinsic risk. Preventable cancer is a subset of cancers with known non-intrinsic causes since to be preventable, a cancer has to have a known and modifiable factor (e.g., Radon is a known factor for lung cancer but not much modifiable.) By the same rationale, preventable cancer is often under-estimated due to the unknown non-intrinsic risk factors

However, this approach inflates the effect of intrinsic factors by assuming there are no other non-intrinsic cancer-causing factors to be identified. Inclusion of a yet to be identified non-intrinsic factor can significantly drive down the contribution of the intrinsic factors as illustrated in Fig. 2. For lung cancer, while Tomasetti et al. estimated the mutation fraction due to intrinsic factors at 33.4%; based on our mutational signature analysis, we identified a much smaller estimate (3.6%) of the intrinsic mutation fraction or a 9-fold difference^15^. This discrepancy could be due to the exclusion by Tomasetti et al. of known exogenous risk factors for lung cancer including radon, a risk prevalent to the entire population and second only to cigarette smoking^33^, as well as second hand smoking and air pollution^34,35^ and yet to be determined environmental and hereditary factors.

### Box 2 Number of driver mutations required for cancer pathogenesis

A stochastic multistage model of carcinogenesis has served as the primary biological theory of cancer for a century. This theory evolved from the early studies of carcinogenicity in animal models and incorporated analysis of cancer incidence in human populations^20,125^. Early work by Yamagiwa and Ichikawa^18^ showed that malignant tumours develop through multiple steps for which, carcinoma development and metastasis were late events dependent on chronic irritation. It was subsequently demonstrated by several groups that tumorigenesis required both exposure to an initiating carcinogen and the presence of tumour promoting factors^16,17^. This initiator/promoter model of tumorigenesis motivated Charles and Luce-Clausen^125^ to posit that the transition from normal cell to early tumorigenesis (papilloma) involved two mutations in a single gene and that carcinogens acted to accelerate the mutation process that would otherwise be rare, i.e., the gene mutation theory. Muller subsequently provided the evidence for the gene mutation theory demonstrating that ionizing radiation, known to be carcinogenic, was also mutagenic. Importantly, the observed latency between radiation exposure and cancer development supported the prevalent hypothesis that more than one mutation per cell was necessary for cancer development^20,21^. Observing an increase in cancer by the sixth power of age, Nordling proposed that cancer may require as many as six consecutive mutations^19^. Building on these works, Armitage and Doll^20^ represented these concepts mathematically as a stochastic multistage carcinogenesis model using a pure birth process finding that the model fit best with six stages analysing the age-specific cancer incidence for several cancers. Subsequently, Knudson^22^ published his two-hit model for retinoblastoma with his theory proven true with the discovery of the retinoblastoma tumour suppressor gene (Rb) in patients with retinoblastoma^23^.

Moolgavkar-Venzon-Knudson (MVK) developed a much used clonal expansion model based on the two successive mutation hypothesis (initiator and promoter) in which they allowed for the possibility that only some cells survive after the first mutation and that cells grow at different rates (semi-stochastic model)^126^. This two-stage model was extended to multiple stages in 1995^24^ in a Frequentist maximum likelihood estimation framework, and more recently to a Bayesian framework^25^.

The earliest effort to estimate the contributions of initiators and promoters on carcinogenesis is attributed to Moolgavkar^127^. In his work, initiator was ‘any’ factor that increased the probability that a normal stem cell would transition to a cell with one hit. A promoter was defined as an agent that promoted the expansion of the ‘intermediate’ cells. Considering age incidence curves, he demonstrated that two cancer risk factors (smoking for lung and oestrogen for breast cancer) modulate tumorigenesis by increasing the transition rate for the promoter rather than the initiator. Analysing the Japanese atomic bomb survivors, Heidenreich and colleagues^26^ extended the multistage model to account for an acute exposure to a mutagen using an age-dependent hazard rate. Indeed, multistage models can be readily extended using discrete or continuous stochastic processes, analytical or numerical methods, to accommodate modern cancer theories.

More recent studies from large-scale sequencing data on cancer genomes suggest that three driver mutations may be sufficient for cancer development for some/most solid tumours^128^. Fewer hits may be required for haematologic malignancies (i.e., cancers of the blood, mostly leukaemias) as bone marrow and blood derived cells need fewer steps to become cancerous, e.g., no requirements for invasiveness and metastatic potential. For example, chronic myelogenous leukaemia (CML) originates with only one mutation^129^, although at this stage CML is a more ‘benign’ cancer, and other mutations are required as CML transitions to a more malignant/lethal phenotype^130^.

## Non-intrinsic risk factors

Mechanisms of non-intrinsic risk factors thought to drive cancers are multifaceted. Some belong to the family of chemicals that induce new mutations (mutagens) while others, such as viruses, induce cancers through activating or repressing key cancer modulating genes (activating oncogenes or inhibiting tumour suppressor genes) in addition to inducing mutations. At least in the case of mutagens, these operate on cells that can divide and persist so as to facilitate tumour development. In defining such ‘at risk’ cell populations, biologic studies have focused on stem cells, progenitor cells, and other dividing cells^36^. In the definition proposed here ‘non-intrinsic factors’ refer to risk factors other than intrinsic replication error, and includes not only exogenous factors (e.g., tobacco, HPV, ultraviolet (UV) radiation, and drugs) but also endogenous factors, such as inflammation, hormones and growth factors, metabolic effects, reactive oxygen species, immune responses, etc. The evidence for non-intrinsic risk factors is mainly derived from studies in cancer epidemiology and cancer biology.

## Exogenous risk factors

Several landmark epidemiological and biological studies have identified exogeneous cancer risk factors such as tobacco smoke for lung cancer, UV radiation for skin cancer, and viruses for cervical and liver cancer. More recently, several groups have reported rising colorectal cancer incidence and mortality rates in Asia approaching those in western countries. Affluent Eastern Asian countries such as South Korea, Singapore, and Japan have experienced a two-fold to four-fold increase in incidence in recent decades^37^. In the USA, a recent study confirmed prior estimates that adults born in 1990 could experience twice the risk of colon cancer and four times the risk of rectal cancer at the same age had they been born in 1950. The reasons for the rise in incidence and death rates remain unclear^38^ but cannot be attributed to change in factors intrinsic to DNA replication machinery in humans and thus, strongly indicate a role for non-intrinsic factors.

### Geographic variation and immigrant studies

**Fig. 3:**
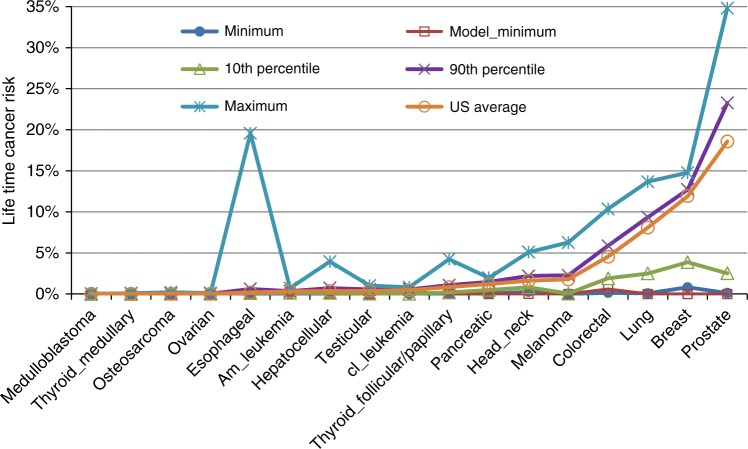
Shown are the (conservative non-zero) minimum, the 10th and 90th percentiles, the US average, and the maximum of the lifetime cancer risk based on World cancer registry, and the stem-cell-model based minimum^6^. The huge disparity between the US average and world minimum indicates that cancer is unlikely the end result of a universal endogenous carcinogenesis mechanism unaffected by exogenous factors (published with permission^6^)

Evidence for causes of cancer in human populations has historically been guided by information on cancer incidence and prevalence rates in different populations. According to GLOBOCAN^39^, incidence rates of different cancers show distinct geographic patterns where estimates in high-incidence regions can be as much as one or two orders of magnitude higher than low-incidence areas. Consistent with this pattern, we recently analysed the World Cancer Registry data^6^ and found that the age-adjusted incidence rates of most cancers show distinct geographic patterns where estimates in high-incidence regions can be as much as ten folds or more than low-incidence areas^6^. Some examples, obtained by taking the ratio of the incidence rates at the 90th percentile and the 10th percentile, include: melanoma (40 fold), colorectal cancer (three fold), lung adenoma (seven fold), breast cancer (three fold), and prostate cancer (nine fold). The difference in world cancer incidence rates and wide disparity are shown in Fig. 3 (originally published in ref.^6^). As shown in this figure, the fold changes will be more dramatic if the ratio is between the regional maximum and minimum.

Favouring environmental risks, seminal work demonstrated that the offspring of immigrants to high incidence regions acquire the incidence patterns of the host country in one or two generations^40^. This adoption of the host country incidence pattern is consistent with changes in factors present in each geographic region. Indeed, higher incidences of lifestyle-related cancers (e.g., breast, prostate, colorectal and lung cancers) have been observed in the early industrialized countries. In contrast, higher incidences of infection-related cancers (e.g., cervical, stomach and liver cancers) have been observed in less developed regions and in areas with endemic infectious agents.

### Retrospective case-control studies

Numerous hypotheses about the role of environmental exposures and cancer have been generated using retrospective case-control studies, in which the association of exposures (e.g., smoking) with cancer can be quantified. Suspecting tarmac or motor car fumes as the major causes for the increased incidence in lung cancer, Doll and Hill^41^ undertook a historical case-control study in 1950. Comparing lung cancer patients with matched controls, they discovered tobacco smoking was strongly overrepresented in the cases. Their findings were subsequently confirmed in a prospective cohort of more than 30,000 British physicians^42^. Over the past several decades, numerous groups have employed the case-control design and odds ratio (OR), under certain assumptions, to estimate the preventable proportion of cancer risk that is attributable to a given exposure: For melanoma, risk ascribed to sun exposure is estimated at 65–86%, and for non-melanoma basal and squamous skin cancers, ~90% is attributable to UV^43^. Here, the attributable risk refers to cancer risk that is theoretically preventable. Additionally, >75% of oesophageal cancer, and >75% of head and neck cancer are attributable to tobacco and alcohol^44,45^ and for the latter, a large fraction of residual risk now suspected to be attributable to HPV^46^. Using this approach, several pathogens (HPV, hepatitis B virus (HBV), hepatitis C virus (HCV) and *H. pylori*) have been identified as cancer causing explaining a majority of cancer of the cervix, liver, stomach and others^47–50^.

### Prospective studies

Supporting links between risk factors and cancer identified in case-control studies, numerous prospective studies have been conducted and proven highly informative. For example, prospective studies on lung^42^, oesophagus and gastric^51^, bladder^52^ and other cancers^53,54^, have confirmed the association of smoking in human carcinogenesis, particularly in the aerodigestive tract. The robustness of the associations has yielded reliable estimates of cancer risk among smokers. Using these estimates and knowledge of smoking rates, the prevalence of smoking-associated cancers has been approximated to be as much as 25–30% of all human cancers^55^.

In the case of cancers associated with infectious agents^56^, prospective studies of *H. pylori* and gastric cancer^57^, HPV and cervical cancer^58^ and recently head and neck cancer^59^, as well as study of HBV and HCV in HCC^60,61^ have yielded evidence linking these agents to tissue-specific cancers. It is currently estimated that infectious agents contribute upwards of 15–20% of all human cancers^56^.

Other physical factors such as ionizing^62^ or UV radiation^63^ contribute causally to cancer incidence, and their linkage to cancer has led to effective preventive measures. High-dose mantle field radiation for the treatment of Hodgkin’s lymphoma was demonstrated unequivocally through prospective studies to increase breast cancer especially those exposed at younger ages^64^. Other sources of ionizing (e.g., environmental radon) and non-ionizing (e.g., UV) radiations have also been linked to lung cancer^65^, leukaemias and lymphomas^66^, melanoma and other skin cancers^63^. These preventable exposures have been estimated to contribute to ~20% of cancers^67^.

In addition to these defined exposures, more complex lifestyle and behaviour factors such as diet, physical activity, alcohol consumption and reproductive patterns have also been intensively studied in cancer risk using the prospective design. For example, physical activity and dietary patterns, particularly nutrient deficient and calorie-dense diets (i.e., high dietary fat, refined sugar, red and processed meats), have been positively associated with high-incidence cancers of modern society including colorectal^68^, breast^69^, prostate^70^ and lung cancer among non-smokers^71^. However, data from prospective studies on specific essential nutrients (i.e., folate, calcium, vitamin D, and others) on cancer risk have been equivocal. The European Prospective Investigation into Cancer and Nutrition (EPIC) study^72^ supports diet as an important or moderately important factor in risk of colorectal and breast but not prostate cancer.

In efforts to increase the sensitivity and reliability between individual dietary factors and cancer, epidemiologists have developed modern analytical methods^73,74^. Employing a meta-analysis of 53 retrospective epidemiologic studies comprising of 58,000 women, women who drank >45 g of alcohol per day were found to have a 1.5-fold higher risk of breast cancer than non-drinkers^75^. This finding was replicated in the Million Women Study in the UK^76^. Using such tools and data, diet has been estimated to contribute to 20–40% of all cancers^77,78^.

While epidemiological studies have a number of strengths, certain inherent weakness limit the reliability of findings when present in only one or a few studies. For geographic comparisons of cancer risks, information on routine medical records and death registries tend to be less accurate or complete in less developed countries and less impacted by asymptomatic, screen detected cancers. This impacts the accuracy and interpretability of the rates. These factors may inflate findings of difference between countries. On the other hand, other considerations may obscure effects of environmental factors. For example, if common exposures exist globally, which may happen increasingly with globalization, it will be harder to recognize their contribution to cancer risk. For retrospective (especially) and prospective study design, confounding effects and selection biases affect the accuracy of the risk and the estimation of the effect size. As such, while replication of findings across studies is among the more powerful criteria for establishing an association, gaining knowledge of the biological mechanisms linking an exposure to disease is a necessary component of the evidentiary process in establishing direct causal relationships.

Despite these inherent limitations, population studies have provided convincing evidence for a major contribution of exogenous factors in cancer risk.

### Mutagens and mutational signatures

Exogenous mutagens, such as UV irradiation, have long been recognized to induce specific mutation patterns in genomes^79^. However, it was not until recently that strong signatures were identified for tobacco^80^ and UV light^81^ in lung cancer and melanoma genomes, respectively. These also provided the proof of principle in discovering the effect of mutagens without knowing their origins. Particularly, capitalizing on many large consortia studies targeting sequencing of large numbers of genomes, such as The Cancer Genome Atlas (TCGA), several mutational signatures have now been identified and characterized with respect to a wide range of cancer types^31,82^. As discussed above (Box 1), this analysis suggests that non-intrinsic factors are dominant in imparting cancer risk. More importantly, given the rapid progress of sequencing technologies, new specific signatures are coming into light with new research that is assigning them to specific exposures. For example, aristolochic acid, common in east Asia and parts of Europe, has been shown to predispose to cancers of the renal pelvis, and is associated with a highly specific signature^83^.

## Endogenous risk factors

Certain cancer risk factors are endogenous to the individual and many have some genetic component. Individual levels of the sex steroid hormones and their role in breast cancer risk are among the best studied examples of an endogenous cancer risk factors^84^. As endogenous determinants of cancer risk, the steroid sex hormones vary over the life course and between individuals and are influenced by other exogenous factors (e.g., diet, therapeutic hormones, other drugs, physical activity levels) as well as other endogenous determinants such as genetic background. Importantly, endogenous sex steroid hormones and cell responses to hormones are proven targets for cancer prevention supporting the modifiability of endogenous risk factors.

More challenging is integrating information on the non-intrinsic effects of complex endogenous processes like ‘ageing’, inflammation, and obesity on cancer risk that are influenced by exogenous (environment) and hereditary (genetic) as an endogenous determinant. For example, obesity has a genetic basis but most often develops as a phenotype from interaction with exogenous factors (over consumption of food and sedentary behaviour) and is thus, highly modifiable. Obesity-associated changes in metabolism, hormones and inflammation are the suspect proximate biological culprits in cancer risk and they are modifiable (metformin, anti-inflammatory drugs, lipid lowering drugs, hormone therapies). Deregulated sex hormones for example are causally linked to the significant increase risk of uterine cancer in obese women^85^. And unlike other cancers, endometrial cancer incidence has continued to increase worldwide^86^ and in parallel with the obesity epidemic^87^. Notable is the reduction (modifiability) of endometrial cancer risk in the obese with weight loss surgery^88^, hysterectomy or use of progestins that oppose oestrogen effects on the endometrial lining^87,88^. In contrast to endometrial cancer, the mechanophysical effects of obesity (i.e., extrinsic gastric compression) in combination with endogenous bile acid reflux into the oesophagus and resultant metaplastic response of the epithelium, explain the rapid rise in oesophageal cancer in the obese—a cancer that was exceedingly rare until the obesity epidemic, and therefore it may be highly preventable.

Here we consider a few such complex endogenous factors and their modifiability. This includes considering ageing as a decline in endogenous anti-cancer processes.

### Inflammation and cancer

From the observations of Virchow and the carcinogenesis studies of Yamagiwa and Ichikawa, an ‘irritation theory’ of cancer was conceived where inflammation was subsequently identified as a major, and in some cancers e.g., asbestos-related mesothelioma and infection-related tumorigenesis, necessary component of malignancy^89–91^. Over the latter half of the 20th century, numerous cellular and molecular mechanism linking inflammation to malignant cell persistence and invasion have been characterized. These range from inflammation-induced reactive oxygen species that act in DNA damage and tumour initiation as well as inflammation-derived cytokine and chemokine effects on tumour growth, angiogenesis and tumour cell migration and invasion^91,92^. Most recently is the appreciation that immune cells play a significant role in suppressing anti-tumour immunity enabling tumour cell persistence and progression to life-threatening disease^93^.

Such effects, and the large body of evidence from animal and human studies, have led to the inclusion of inflammation as an enabling factor to carcinogenesis^94^, where inflammation is accepted to act across the continuum of tumorigenesis in a number of cancer types. The significance of inflammation in cancer development has been demonstrated in the chemoprevention field where randomized clinical trials and population studies of anti-inflammatory agents such as aspirin and other non-steroidal anti-inflammatory drugs have demonstrated the cancer prevention effects of suppressing pro-inflammatory mediators like prostaglandin E2 for several cancers^95^. Indeed, in 2015 the US Prevention Services Task Force recommended in favour of low dose aspirin use for the prevention of colorectal cancer in individuals at elevated risk that include patients with Hereditary Non-Polyposis Colorectal Cancer (HNPCC) Syndrome who carry germline mutations in mismatch repair genes^96^.

While it is clear that inflammation is critical for tumour development, incorporation of inflammation in mathematical models of tumour development is lacking. This stems in part from the lack of valid biomarkers of cancer associated inflammation. As with mutational signatures of carcinogens, and more recent efforts to assess ageing, integration of inflammatory signals with the genomic and sequence data may offer insights on the magnitude of cancer burden that can be attributed to inflammation—work that would greatly enhance efforts aimed at modifying inflammation as a prevention strategy for reducing cancer incidence in the population.

### Ageing

Ageing is considered among the most significant risk factors for cancer^97^. Yet, it is important to recognize that ageing can be defined chronologically or biologically. Chronological ageing contributes toward cancer by providing time for intrinsic risk as well as for exogenous and endogenous factors including mutagens to exert their effects. In contrast, biological ageing processes are more difficult to define or quantify since their full spectrum is not fully understood. The strong positive association of ageing with cancer is widely believed to reflect generalized declines in cellular and molecular system functions as an endogenous risk. Ageing encompasses at least nine recently proposed hallmarks^98^ for which there are numerous overlaps to the cancer hallmarks^94^: genomic instability, telomere attrition, epigenetic alterations, loss of proteostasis, deregulated nutrient sensing, mitochondrial dysfunction, cellular senescence, stem cell exhaustion, and altered intercellular communication.

In an effort to assess the impact of ageing, Podolskiy et al.^99^ reported that accumulation of age-associated CT and GA mutations at CpG sites (common replication errors) appears to accelerate in a monotonic fashion until later in life (50–80 years) when the rate declines. The group reports that that the acceleration in mutation burden is higher in men and initiates earlier in life in men. This parallels higher overall cancer incidence in men and an earlier age (about 10 years) at which cancer incidence begins to rise in men. The authors suggest that the strong representation of age-associated mutations in tumours reflect decreases in organismal fitness with ageing that differ by gender and tissue type.

Not all biological ageing is pro-tumorigenic. For example, mechanistic studies have suggested that cell senescence and stem cell exhaustion that accelerate with ageing may explain the observed decline in incidence of cancer at the extremes of human age^100,101^. It is worth mentioning that the rate and peak timing of age-related cancer risk varies from cancer to cancer and even within subgroups of specific cancers. This suggests that there is not always a positive relationship between age and cancer risk. For breast cancers, triple negative and HER2 positive breast cancers peak earlier in adulthood and exhibit a decline with advancing age where oestrogen receptor positive breast cancer incidence rises later and continues to increase with age^102^. These results may also reflect differences in susceptible cell populations in tissues that senescence at different life-stages; life cycle biology not currently considered in modern models of cancer risk.

To tease out ageing effects on cancer from non-intrinsic risk factors is challenging. The effect of ageing on cancer risk is commonly removed by testing the cancer risk in individuals with and without the exposure matched on age. Analysis of age-adjusted incidence rates provides the most common way to address this issue. In the geographic comparisons discussed previously, all the incidence rates are age-adjusted. In these cases, the chronologic age effect is accounted for.

### Heritable factors

Hereditary cancer can also operate through intrinsic and non-intrinsic mechanisms, by modulating the frequency of mutations per se (or their repair) but also by non-intrinsic mechanisms. For example, for cancers of the breast, prostate and colon, upwards of 30–40% of the attributable risk of these cancers is thought to be due to genetic causes. A landmark paper published in 2000 of 11 cancer types in 44,788 twin pairs concluded in favour of the environment as an “overwhelming contributor to the causation of cancer”, a statement that, like current discussions, prompted vigorous debates^103^. Importantly however, the work provided evidence of significant heritability in the common cancers of prostate (42%), colorectum (35%) and breast (27%). In a recent study of 80,309 monozygotic and 123,382 same-sex dizygotic twins of the Nordic Twin Study of Cancer (NorTwinCan)^104^, a 33% excess familial risk was observed for all cancer with confirmation that the magnitude of excess heritable risk was cancer specific with nearly 60% of prostate cancer estimated to be influenced by genetic factors. While much of the genetic basis of cancer risk remains to be identified, it is notable that a majority of the hereditary cancer mutations as well as the germ line variants identified involve DNA repair genes thought to act by increasing mutation rates often in tissue-specific fashion. However, it is also important to note that the increased risk may also derive from genetic mechanisms resulting in increased susceptibility to non-intrinsic factors and exposures to other DNA damaging processes.

### Tumour epigenetics

Our ability to understand and to model cancer aetiology and the impacts of exogenous and endogenous factors in risk has in recent years been extended to include consideration of effects of numerous factors on the epigenome. As with replication errors, epigenetic changes (e.g., DNA methylation) are passed on to daughter cells as non-sequence based chemical changes to the DNA. There is convincing evidence that epigenetic changes not only occur during tumour development, but they also play a direct causal role. This includes reproducible evidence that specific epigenetic silencing events, such as silencing of MLH-1 in a subset of human colon cancers, are essential alterations in human tumorigenesis^105^. Key epigenetics mechanisms in human carcinogenesis are beyond the scope of this perspective but have recently been reviewed for the major cancer types^106^. Noteworthy here for future models aimed at (1) identifying cancer risk factors and (2) for estimating contribution of factors (endogenous or exogenous) that impact the epigenome in cancer risk is consideration of the recent elegant work from the Baylin laboratory^107^. In their studies, they provide evidence that cigarette smoke (as a chronic exposure) induces time dependent alterations in the human bronchial epithelial cell epigenome that enhances their sensitivity to transform with a single KRAS mutation^107^. These data strongly suggest that a chronic exposure like smoking (or obesity, nutrient deprivation, ageing epigenetic effects on immune cell function, inflammation) may act by lowering the threshold of a cell to intrinsic errors for cancer development; an important interaction of the effects of the intrinsic and non-intrinsic risk factors not adequately considered in previous models. Similar effects of other exogenous and endogenous factors to the epigenome including inflammation, obesity and ageing may similarly alter the thresholds to transformation via effects on the epigenome^108,109^. Importantly, whether epigenetic changes represent reversible processes is currently debated and a subject of investigation. Studies in smokers, however, demonstrate smoking-specific changes to the epigenome persist for many years after smoking cessation, which may explain the long-lasting nature of elevated risk in former smokers.

### Other endogenous factors

In addition, less well defined ‘endogenous’ factors such as complex gene × gene interactions and gene × environment trait interactions are increasingly recognized as ‘cancer risks’. These include height and telomere length as examples along with emerging interest in the human microbiome as a modifier of cancer risk. Given progress toward understanding the significance of complex interactions in cancer, estimating their contribution to cancer burden will be important. While beyond the scope of this review, two recent lines of work on telomere genetics and cancer risk and human height and cancer risk are worth mentioning^110^.

The Telomeres Mendelian Randomization Collaboration Group^110^ recently demonstrated an association between genetic polymorphisms, telomere length and cancer. Longer telomere associated gene variants were associated with rare cancers and strikingly, with cancers in tissues with low stem cell divisions. As noted by the authors, the positive association with telomere length is consistent with evidence that telomere shortening with aging may act as an intrinsic protective mechanism against cancer by limiting cell division, explaining the lower rates of cancer in extreme age. While telomere length is a heritable trait, recent evidence from experimental models suggests that telomere length is malleable and influenced by numerous external stimuli^111^. Such findings provide new biological rationale for positive associations between environmental and psychosocial factors and telomere length observed in human studies that may impact cancer risk^112^.

Similarly, the repeated observation between adult height and cancer risk^113^ including breast^114^, prostate and colon^115^ is intriguing given the average height of humans continues to increase worldwide. Height is a heritable trait with estimates from twin studies suggesting that as much as 80% of height, especially in adolescence, can be attributed to parental height^116^. As such, the positive association between height and cancers has been hypothesized to reflect genetic traits that influence gestational, childhood and adolescent growth processes that also act on cancer progenitor cells. Indeed, 168 genetic variants associated with height and Mendelian randomization analysis were reported associated with genetically predicted height and risk of oestrogen receptor positive breast cancer^114^. Confounding the interpretation of these associations though is the strong influence of maternal nutrition as an equally strong non-genetic determinant of height^117^. Such important gene × environment interactions may partly explain geographical differences in risk of certain cancers like prostate cancer. For example, prostate cancer has been shown to be positively associated with height at 13 years of age^118^; a time when early life nutrition is most important in determining stature. This association was independent of adult height, suggesting nutrition in early life may be a modifying factor in prostate cancer risk. Like emerging evidence that obesity and other growth factor affect cancer risk via expansion on tissue stem cells^119^, it is plausible that nutrition and height genes interact with effects on stem cells affecting an intrinsic risk factor for cancer at the tissue level. Understanding such effects will be essential for modelling the contribution of each to cancer risk. Unfortunately, integration of early life exposures including nutritional status in human studies are challenging and make it difficult to tease out the effects of early life nutrition on adult cancer^120^. Studies in animals and in birth to death cohorts, where detailed early life exposures are collected, will be critical to advancing our understanding of such factors in risk of cancer in adults^121^.

## Conclusions and perspective

**Fig. 4:**
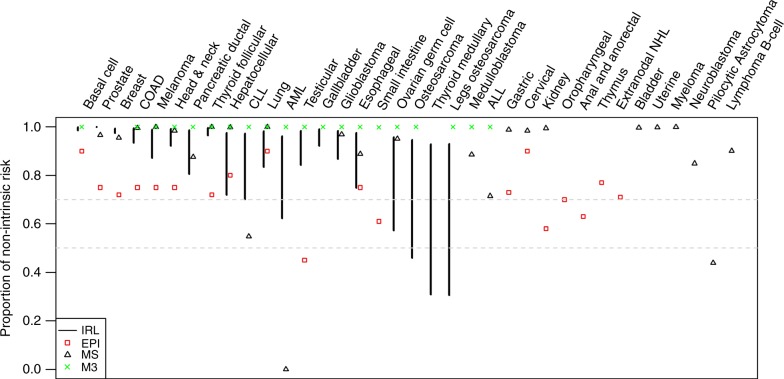
Proportion of non-intrinsic risk estimates from four different approaches. Data were obtained from ref ^15^. The two dashed horizontal lines indicate non-intrinsic risk at the levels of 0.5 and 0.7. IRL confidence interval from the intrinsic risk line method, EPI epidemiological estimates, MS estimates based on mutational signatures (Box 1), M3 estimates from the 3-hit model, AML: Acute Myeloid Leukemia, ALL: Acute Lymphocytic Leukemia, CLL: Chronic Lymphocytic Leukemia, NHL: Non-Hodgkin’s Lymphoma. Most cancers show substantial non-intrinsic risks, except for AML, ALL, CLL and Pilocytic Astrocytoma, all of which are rare cancers and contribute less than 1% of the total cancer burden, and therefore those results do not affect our overall estimates. Moreover, AML, ALL and CLL are blood cancers whose pathogenesis and requirement for mutations may differ from solid tumours

Multiple approaches have been applied over the past few decades to understand and determine cancer exposures and risks (Boxes 1–2). These have aided in mathematical modelling approaches aimed at estimating the contributions of non-intrinsic and intrinsic factors to cancer risk and cancer burden in the population. Overall, except for a few isolated studies^7,8^, for most cancers, estimates from various approaches attribute a sizable fraction of cancer risk (60–90%) to non-intrinsic risk (Fig. 4). These estimates of non-intrinsic risk are consistent across studies and support a substantial contribution of potentially modifiable or actionable risk to cancer^77,78,122^. Evolving theories in cancer molecular pathogenesis and technological innovations (for example the deeper understanding of the cancer epigenetics mechanisms) are resulting in finer estimates of the impact of intrinsic and non-intrinsic processes based on biological principles. The rapid advances in the molecular biology of human cancers, including emergent role of stem cells in cancer evolution and expansion of long lived clones with multiple mutations and epigenetic changes, favour a much more complex picture of cancer aetiology with heterogeneity among the cancers and within cancers of the same tissue type. These pave the way for development of new analytic approaches that better integrate new knowledge including considering contributions of individual factors as well as their joint effects on cancer burden.

Much discussion has been made recently of the role of ‘bad luck’ in cancer risk, where the contribution of intrinsic factors to cancer is considered unmodifiable bad luck^7,8^. Thus, someone who never smoked may still have a lifetime risk of lung cancer of 0.2% to 1%. However, it is important to realize that (1) Non-intrinsic factors themselves only impart an increase in risk in developing cancer, and therefore there is still an element of luck for non-intrinsic factors. For example, whether a smoker develops lung cancer or not, an event that has a lifetime probability of 10–25%, depends on other factors including their sex and degree of smoking. Smoking increases the probability by 10 to 25 folds. Thus, exposure to risk factors does not necessitate the development of cancer; nor does the absence of exposure (with a few exceptions e.g., HPV) provide a 100% guarantee to prevent cancer. (2) Non-intrinsic and intrinsic risk factors often do not act independently as we have highlighted, and the most likely scenario is that they cooperate to cause cancer. In this regard, cancer risk can still be modified even when intrinsic factors contribute to some of the risk. As such, for some cancers there is evidence that there is an ‘unmodifiable’ variation arising from the built-in randomness of intrinsic and non-intrinsic mechanisms and this is likely greater in tissues with a higher level of cell division.

As such, it is detrimental to prevention and cancer control measures if the risk, especially for clinically significant cancers, is over interpreted to be due solely to bad luck. This underestimates the potential impact of prevention and control measures aimed at reducing or delaying incidence and death due to cancer. Similarly, under-estimating the fraction of preventable cancer risk impedes progress to identify modifiable exposures for cancer prevention and control measures when possible (Fig. 2).

Indeed, the proportion of currently preventable cancers is mostly a subset of cancers with known non-intrinsic risk factors (as shown in Fig. 2). Per the Cancer Research UK, ~40% of cancer burden is currently preventable. For example, at present, several cancers (e.g., prostate, thyroid, brain and testicular cancers) show no benefit from the modification of 14 lifestyle and environmental risk factors^123^ even though epidemiologic and other studies suggest strong effects of the environment. Therefore, this does not negate the significant contribution of currently unidentified risk factors or that they would become modifiable. Moreover, other known non-intrinsic risk factors such as radon for lung cancer and geographic variations for breast, colorectal and prostate cancers are not currently considered in the Cancer Research UK estimates. While plausible, challenges remain in ascertaining exposure of humans to putative non-intrinsic risks with hypothesized but equivocal evidence for several suspects, including heavy metals, endocrine disruptors, cadmium, sleep deprivation, chemical mixtures especially at low doses and nutrient deficiencies (folate, selenium) identified from experimental systems as pro-tumorigenic.

Potential interactions among various risk factors further complicates measurement issues, though the identification of additional modifiable risk factor(s) will likely open new venues for prevention (or at least intervention). This has been amply illustrated with the successive discovery of risk factors such as smoking, HPV, inflammation in colon cancer, and many others. With modern knowledge, there are also prevention successes in several hereditary cancers. For example, knowledge of the effects of BRCA1 mutation (an endogenous risk) on biological process has facilitated primary prevention including removal of the ovaries to reduce risk of breast cancer and benefits of tamoxifen; findings that support hormone modifying effects on endogenous risk that are modifiable. Similar concepts for the effects of aspirin in families with Hereditary Non-Polyposis Syndrome, a mismatch repair gene deficiency that increases mutation rates are likely to advance prevention efforts aimed at modifying intrinsic and other endogenous processes like ageing.

From our perspective, critical challenges going forward in understanding cancer risks include the need to continue to advance the biological understanding of cancer causation. Importantly, this includes the modern challenge of defining and distinguishing significant cancers (i.e., those that pose risk to life and significantly impact patient well-being) from those that do not and determining to what degree the attributable risk is preventable. Moving forward we need to establish comprehensive sequencing databases on both high and low-incidence regions for major cancers, and link biological theories with observed/experimental data through enhanced modelling and analysis efforts with more concerted efforts to advance models that deal with the complexity of cancer aetiology including simulating the joint effects of intrinsic and non-intrinsic risk factors.
